# Reporting the national antimicrobial consumption in Danish pigs: influence of assigned daily dosage values and population measurement

**DOI:** 10.1186/s13028-016-0208-5

**Published:** 2016-05-03

**Authors:** Nana Dupont, Mette Fertner, Charlotte Sonne Kristensen, Nils Toft, Helle Stege

**Affiliations:** 1 Department of Large Animal Sciences, University of Copenhagen, Grønnegårdsvej 2, 1870 Frederiksberg C, Denmark; 2 National Veterinary Institute, Technical University of Denmark, Bülowsvej 27, 1870 Frederiksberg C, Denmark; 3 SEGES Pig Research Center, Vinkelvej 13, 8620 Kjellerup, Denmark

**Keywords:** Animal daily dose, Antibiotics, Antimicrobials, Pigs, Surveillance

## Abstract

**Background:**

Transparent calculation methods are crucial when investigating trends in antimicrobial consumption over time and between populations. Until 2011, one single standardized method was applied when quantifying the Danish pig antimicrobial consumption with the unit “Animal Daily Dose” (ADD). However, two new methods for assigning values for ADDs have recently emerged, one implemented by DANMAP, responsible for publishing annual reports on antimicrobial consumption, and one by the Danish Veterinary and Food Administration (DVFA), responsible for the Yellow Card initiative. In addition to new ADD assignment methods, Denmark has also experienced a shift in the production pattern, towards a larger export of live pigs. The aims of this paper were to (1) describe previous and current ADD assignment methods used by the major Danish institutions and (2) to illustrate how ADD assignment method and choice of population and population measurement affect the calculated national antimicrobial consumption in pigs (2007–2013).

**Results:**

The old VetStat ADD-values were based on SPCs in contrast to the new ADD-values, which were based on active compound, concentration and administration route. The new ADD-values stated by both DANMAP and DVFA were only identical for 48 % of antimicrobial products approved for use in pigs. From 2007 to 2013, the total number of ADDs per year increased by 9 % when using the new DVFA ADD-values, but decreased by 2 and 7 % when using the new DANMAP ADD-values or the old VetStat ADD-values, respectively. Through 2007 to 2013, the production of pigs increased from 26.1 million pigs per year with 18 % exported live to 28.7 million with 34 % exported live. In the same time span, the annual pig antimicrobial consumption increased by 22.2 %, when calculated using the new DVFA ADD-values and pigs slaughtered per year as population measurement (13.0 ADDs/pig/year to 15.9 ADDs/pig/year). However, when based on the old VetStat ADD values and pigs produced per year (including live export), a 10.9 % decrease was seen (10.6 ADDs/pig/year to 9.4 ADDs/pig/year).

**Conclusion:**

The findings of this paper clearly highlight that calculated national antimicrobial consumption is highly affected by chosen population measurement and the applied ADD-values.

## Background

In recent years there has been an increasing concern towards the occurrence of antimicrobial resistance in both human and veterinary pathogens. This has led to a rise in the monitoring of veterinary antimicrobial usage [1] enabling detailed reports on antimicrobial consumption levels [2–5]. To minimize misinterpretations due to calculation method, it is crucial that reports on antimicrobial consumption are easily understandable and transparent [6, 7], especially when evaluating consumption over time and when comparing different animal populations—e.g. different countries [2, 8, 9].

In Denmark, detailed data on veterinary antimicrobial consumption from the national database VetStat [10] are summarized and published in yearly DANMAP-reports and on the Danish Veterinary and Food Administration’s (DVFA) webpage [11, 12]. Furthermore, DVFA draws up monthly reports on pig antimicrobial consumption at herd level in conjunction with the antimicrobial restrictive legislation, known as the Yellow Card initiative [13]. DANMAP and DVFA both report antimicrobial consumption using the measurement unit “Animal Daily Dose” (ADD) [14, 15]. Previously, both DANMAP and DVFA used the same set of standardized values for weight at treatment and dosage per kg body weight (ADD-value) when calculating the consumption as number of ADDs. The ADD-values were assigned at product level in VetStat and based on the approved dosage in the summary of product characteristics (SPC), but in principle adjusted so the same quantity of active compound, concentration and administration route resulted in the same ADD count [15]. In 2011, new products emerged with a considerably higher SPC approved dosage compared to identical competing products. Due to substantial differences in SPC approved dosages, the previous standardization in VetStat was not possible. These products’ ADD-values in VetStat were then based solely on the dosage value stated in the SPC. Consequently, products with the highest SPC dosage value were favored on the market as they resulted in a lower ADD count at herd level compared to similar products. This created a need for non-SPC based ADD-values to eliminate bias when evaluating the true resistance selective pressure. A new set of ADD-values was therefore introduced in the DANMAP 2012-report. The new DANMAP ADD-values were based solely on active compound, concentration and administration route [16]. Later in spring 2014, DVFA also introduced a new set of ADD-values, which was implemented on the 30th of November 2014 and applied in the Danish Yellow Card initiative [17].

To take the population at risk into account when reporting the antimicrobial consumption, DANMAP uses both data on number of produced animals and data on number of live animals present [12, 18] and DVFA uses data from the Central Husbandry Register, which keeps records on number of pigs registered in each herd [19]. The chosen population measurement may affect the calculated antimicrobial consumption [20, 21]. This is especially true for Denmark, which has experienced a large shift in production pattern. In 2000, Denmark produced 22 million pigs of which 6 % were exported live. Through 2007 to 2013, the production of pigs increased from 26.1 million pigs per year with 18 % exported live to 28.7 million with 34 % exported live [22, 23]. Of the exported pigs in 2013, 91.9 % weighed approximately 30 kg at export [23]. In 2012, 7–30 kg pigs were reported to consume 77 % of the total Danish pig antimicrobial consumption calculated in number of ADDs [24]. Excluding the exported live pigs when summing up antimicrobial consumption per produced pig might therefore lead to skewed results.

Several papers have investigated the consequences of using different measurement units when reporting antimicrobial consumption [3, 7, 25]. Additionally, a paper was recently published on how the calculated Dutch pig antimicrobial consumption in 2012 was affected by using three different sets of ADD-values [26]. However, to our knowledge no paper has yet been published which both describes how choice of population measurement and set of ADD-values affect findings when evaluating the national veterinary antimicrobial consumption over time.

The aims of this paper were therefore (1) to describe the previous and present methods used by two major Danish institutions to assign ADD-values and (2) to illustrate how differences in choice of population and assigned ADD-values affect the calculated national pig antimicrobial consumption in the years surrounding the introduction of the Yellow Card initiative (2007–2013).

## Methods

### The study was performed as a retrospective database study

#### Description of previous and present ADD-values

The three sets of ADD-values were collected from the relevant sources. The old VetStat ADD-values were extracted directly from VetStat on the 31st of March 2014. The new DANMAP ADD-values, applied in the DANMAP 2012 report, were collected from The National Food Institute, Technical University of Denmark (DTU) and the new DVFA ADD-values were downloaded from DVFA’s webpage (https://vetstat.dk) on the 30th of December 2014. Only ADD-values for pigs were investigated. The three sets of ADD-value were compared and subjected to descriptive analyses to identify differences and similarities. Both ADD-values according to DANMAP and DVFA may change over time as new products are added and other changes are made. The presented results in this paper therefore solely represent a snapshot in time.

#### Presenting antimicrobial consumption based on four different population measures and three different sets of ADD-values

##### Pig population measurements

To investigate how the chosen population affected the calculated national antimicrobial consumption, the Danish pig population was estimated according to the four following population measurements:

1. Number of pigs according to Statistics Denmark (SD). SD estimates the pig population in four quarterly surveys based on questionnaires from a random sample of 2500 pig herds [27]. The numbers are available to the public on SD’s webpage. SD numbers are thought to represent live pigs present in the Danish herds at that particular point in time.
2. Number of pigs according to The Central Husbandry Register (CHR). This national database holds registrations on “*number of animals per age group present in the herd under normal circumstances*” [28]. Larger pig herds (>300 sows, ≥3000 finishers and/or 6000≥ growers) are required to approve or update data on number of animals per herd minimum twice per year, while all other herds are required to approve or update data to CHR a minimum once a year [29]. Data from CHR are used by DVFA in the Yellow Card initiative.
3. Number of pigs slaughtered in Denmark per year (SL-year). This number is published annually by the Danish Agriculture and Food Council [30].
4. Number of pigs produced per year (PROD-year). This number includes number of pigs slaughtered per year in Denmark and the number of live exported finishers, breeding gilts, sows and growers (exported at approximately 30 kg) and is published annually by the Danish Agriculture and Food Council [22, 23].

##### Calculation of antimicrobial consumption

Data on pig antimicrobial consumption from the 1st of January 2007 to the 31st of December 2013 were collected from the national database VetStat. The VetStat data extraction was made the 31st of March 2014. VetStat contains detailed data on all veterinary drugs sold. A data entry in VetStat pertaining to a purchase of an antimicrobial product for use in production animals always contains: date of purchase, product purchased, amount of product, herd identification code and which age group and disease group the product has been prescribed for [10]. Data entries on pig antimicrobial consumption submitted by both pharmacies, veterinarians and feed mills were included for the whole period (a total of 1887,732 entries). Data entries from VetStat on antimicrobial purchase with a missing or invalid age group were excluded from the study (0.36 %: 6770 entries).

The national annual pig antimicrobial consumption was calculated in number of ADDs. To calculate number of ADDs the following must be known: quantity of product, dosage of product per kg body weight and the weight of the animal at treatment. Expected weight at treatment was set using the same standardized VetStat-values as both DANMAP and DVFA apply: growers (15 kg), finishers (50 kg) and pre-weaning pigs, sows, boars and gilts (200 kg). For dosage of product per kg body weight, the three collected sets of ADD-values were each applied—from VetStat (old VetStat ADD-values), DTU (new DANMAP ADD-values) and DVFA (new DVFA ADD-values).

Number of ADDs was calculated by using the same formula as VetStat, DANMAP and DVFA:

$$ ADD = \frac{{{\text{Amount of product sold}}^{a} }}{{{\text{dosage \,\,pr\,\, kg\,\, body\,\, weight}}^{b} \times {\text{standard\,\, weight}}^{c}  }} $$

a: antimicrobials registered per year for use in pigs according to VetStat data. b: ADD-value according to either VetStat, DANMAP or DVFA. c: standardized VetStat-values for weight at treatment: growers (15 kg), finishers (50 kg) and pre-weaning pigs, sows, boars and gilts (200 kg).

Knowing the number of ADDs sold in a year, it was then possible to estimate number of ADDs per pig per year in relation to the four measurements for pig population: SD, CHR, SL-year and PROD-year.

For SD, CHR and SL-year, the total amount of antimicrobials recorded for use in pigs in VetStat was used when calculating ADDs/pig/year. However, for PROD-year, we needed to adjust the total consumption according to VetStat with an estimate of the extra amount of antimicrobials that were expected to be used, had these growers remained in Denmark. Firstly, not all exported growers would have survived until slaughter. We used an expected mortality of 3.8 % (average finisher mortality 2007–2013 [31]) and reduced the number of would-have-been finishers accordingly. Secondly, we calculated the average antimicrobial usage in the finishing period in Denmark by dividing both total kg active compound and total number of ADDs (used for pigs >30 kg) with the number of pigs slaughtered per year in Denmark (i.e. calculating average usage per finisher/year). Thirdly, we multiplied the adjusted number of exported growers (minus the 3.8 %) with the average finisher antimicrobial usage and added this extra amount to the actual annual consumption as reported from VetStat.

## Results and discussion

### Description of the previous and present ADD-values

Both VetStat, DANMAP and DVFA have defined ADD as the assumed average maintenance dose per day for the main indication in a specified species [15, 18, 19]. The old VetStat ADD-values were based on the SPCs. In contrast, the new DANMAP ADD-values and the new DVFA ADD-values were solely based on active compound, concentration and administration route [16, 19]. Despite seemingly identical theoretical foundations when determining a product’s ADD-value, discrepancies between the new DANMAP ADD-values and the new DVFA ADD-values were observed.

VetStat listed ADD-values for 660 antimicrobial products for use in pigs, which included products intended for both oral, parenteral and intrauterine administration. DVFA listed ADD-values for 666 antimicrobial products for use in pigs, including products for parenteral, oral and intramammary use. DANMAP listed ADD-values for 636 antimicrobial products for pigs, including products approved for oral or parenteral use. DANMAP did not list ADD-values for intramammary or intrauterine antimicrobial products.

ADD-values stated by both DVFA and DANMAP were only identical for 48 % (309/648) of the antimicrobial products approved for use in pigs. The mean percentage difference for the 339 products with unequal ADD-values was 21.8 % (std. dev.: 21.1; median: 20). This discrepancy between ADD-values, despite a seemingly identical theoretical foundation, may be due to the fact that DVFA has used “dosage for the most frequently used indication” as a starting point when deciding ADD-values [19], whereas DANMAP has used the dosage closest to the ones recommended in “The Veterinary Formula” published by the British Veterinary Association in 2005 [16].

Compared to the old VetStat ADD-values, 30.5 % of the products had been assigned a new ADD-value by DVFA (203/666). The mean percentage difference for the 203 products with unequal ADD-value was 32.8 % (std. dev: 33.4; median: 25). A few examples of products with differing ADD-values are shown in Table 1.

**Table 1:** Example of products with changed ADD-value

Product name	Active compound	Concentration	Gram or mL product per kg live weight (ADD value)					
			*Old VetStat*	*New DANMAP*		*New DVFA*		
			Actual ADD	Actual ADD	Change from old VetStat (%)	Actual ADD	Change from old VetStat (%)	Change from DANMAP (%)
Lincomix vet	Lincomycin	110 mg/g	0.044	0.0454	+*3*	0.11	+*150*	+*142*
Aivlosin	Tylvalosin	8.5 mg/g	0.25	0.5	+*100*	0.25	*0*	+*100*
Aquacycline vet	Tetracycline	180 mg/mL	0.04	0.0416	+*4*	0.056	+*40*	+*35*
Denagard vet	Tiamulin	125 mg/mL	0.14	0.069	−*51*	0.072	−*52*	+*4*
Ladoxyn	Doxycycline	500 mg/g	0.04	0.025	−*38*	0.02	−*50*	−*20*
Suprim vet	Sulfa-TMP	120 mg/mL	0.21	0.25	+*19*	0.2	−*4.8*	−*20*

### Changes in the Danish pig population

**Fig. 1:**
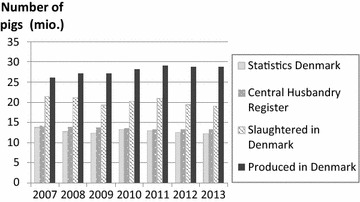
The annual Danish pig population. From 2007 to 2013 according to (1) Statistics Denmark’s annual summer survey, (2) the Central Husbandry Register on the 31st of December in the corresponding year, (3) pigs slaughtered in Denmark per year and (4) pigs produced in Denmark per year

Through 2007 to 2013, PROD-year was approximately twice as high as SD and CHR (Fig. 1). This is as expected as the average time from birth till slaughter is approximately 5–6 months. In the same time span, the Danish pig production increased by 10.3 % from 26.1 to 28.7 million pigs per year when measured as PROD-year (Fig. 1). The difference between SL-year and PROD-year was caused by a shift in production pattern. From exporting 18 % of produced pigs live in 2007, 34 % were exported in 2013. This increase in live pig export was solely driven by a 161 % increase in the export of live growers (3.5 million in 2007 to 9.2 million in 2013), as the export of live finishers and sows in the same time span decreased by 61 and 66 %, respectively (finishers exported: 2007 899,439; 2013 350,447; sows exported: 2007 203,827; 2013 72,245). Through all years, number of sow slaughtered remained between 43,000 and 51,000.

### Presenting antimicrobial consumption based on four different pig population measurements

From 2007 to 2013, the antimicrobial consumption, measured as total number of ADDs per year, increased by 9 % when using the new DVFA ADD-values (278–303 million ADDs). However, the total consumption decreased by 2 and 7 % respectively when using the new DANMAP ADD-values (280–273 million ADDs) and the old VetStat ADD-values (266–247 million ADDs), respectively.

**Fig. 2:**
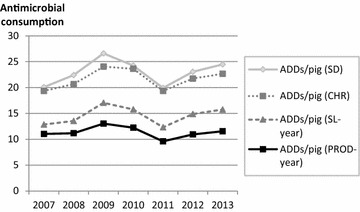
Annual antimicrobial consumption using four different measurements for the pig population. Number of Animal Daily Doses (ADDs)/pig/year calculated using the new DVFA ADD-values and the following four measurements for the pig population: (1) Statistics Denmark’s annual summer survey (SD), (2) number of pigs registered in the Central Husbandry Register on the 31st of December in the corresponding year (CHR), (3) pigs slaughtered in Denmark per year (SL-year) and (4) pigs produced in Denmark per year (PROD-year)

Figure 2 illustrates how the chosen population measurement affects the calculated national average antimicrobial consumption per pig.

When calculating the antimicrobial consumption using SL-year and the new DFVA ADD-values, the consumption increased by 22 % from 2007 to 2013, whereas during the same time span the consumption decreased by 4.5 % when using PROD-year as population measurement. When the new DVFA ADD-values were applied, the national average antimicrobial consumption per pig was approximately twice as high when using SD or CHR as population measurements compared to PROD-year (e.g. in 2011: SD 20.0 ADDs/pig/year; CHR 19.5 ADDs/pig/year; PROD-year 9.6 ADDs/pig/year). In other words, the estimated number of standardized treatments per pig per year was twice as high when using number of pigs according to SD or CHR as when using PROD-year. It is not surprising that ADDs/pig/year based on PROD-year is comparably lower than when based on SD or CHR, as the number of pigs produced in a year will naturally be higher than the number of pigs present at one single point in time. One could argue that (i) SD or CHR and (ii) SL-year or PROD-year should never be directly compared, as they represent fundamentally different ways of tallying up the pig population. However, the differences are illustrated in this paper to underline the necessity of clearly disclosing which population is used and illustrate how the choice can affect calculated results on antimicrobial consumption. In addition, it should here be underlined that ADD is strictly a theoretical unit, which is not necessarily reflective of the actual number of dosages used, as illustrated in previous studies [7, 25].

Based on these findings, it is evident that including or excluding live exported pigs highly affects the calculated results when estimating the national average antimicrobial consumption per pig. This especially holds true in a country such as Denmark, where a substantial part of the pigs are exported live after having reached 30 kg. Consequently, these pigs may have spent the period where they are most likely to require the majority of their antimicrobial treatments in Denmark [24]. Not including the live export may lead to potentially faulty conclusions when estimating the national average pig antimicrobial exposure, as this calculation will be based on the assumption that all antimicrobials were consumed by the remaining pigs which were slaughtered nationally. Choice of population is also highly relevant when comparing antimicrobial consumption across borders. It is critical that researchers and other stakeholders take production demographics into account when reporting antimicrobial consumption, especially when comparing countries, such as Denmark or the Netherlands, with a large export of live growers, to countries with a large import of live pigs, such as Germany and Poland, or to countries which neither have a large import nor export, such as e.g. Sweden [32].

### Presenting antimicrobial consumption based on three different sets of ADD-values

**Fig. 3:**
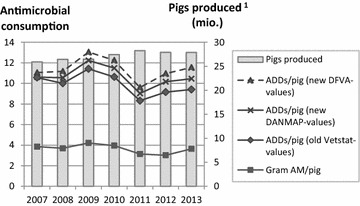
Annual antimicrobial consumption using three different sets of ADD-values. Number of Animal Daily Doses (ADDs)/pig/year calculated using PROD-year as population measurement and the following three sets of ADD-values: (a) the old VetStat ADD-values used in the Yellow Card initiative until the 29th of November 2014, (b) the DANMAP ADD-values used in the 2012 and 2013 DANMAP reports and (c) the Danish Veterinary and Food Administration’s ADD-values used in the Yellow Card initiative from the 30th of December 2014 and onwards. ^1^Pigs produced adjusted for an assumed 3.8 % mortality in exported growers

Figure 3 illustrates how the chosen set of ADD-values affects the calculated national average antimicrobial consumption per pig. If the consumption was calculated as gram active compound, number of ADDs using the old VetStat ADD-values or number of ADDs using the new DANMAP ADD-values with PROD-year as population measurement, a reduction was observed in the average antimicrobial consumption per pig from 2007 to 2013 (5.6, 10.9 and 1.6 % respectively). However, when using the new DVFA ADD-values, antimicrobial consumption per pig per year increased by 4.5 % during the same time span. From 2011 and onwards, an increasing difference in the calculated consumption could be observed between the three different sets of ADD-values. When using PROD-year as population measurement, the consumption was 15 % higher in 2011 when using the new DVFA ADD-values (9.6 ADDs/pig/year) compared to the old VetStat ADD-values (8.3 ADDs/pig/year). In 2013, the calculated consumption was 23 % higher when using the new DVFA ADD-values (11.6 ADDs/pig/year) than when using the old VetStat ADD-values (9.4 ADDs/pig/year). This increasing difference may have been caused by a shift towards purchase of products which gave a low number of ADDs on paper and the release of several products with a higher approved dosage in the SPC compared to competing, similar products.

### Presenting antimicrobial consumption based on four different pig population measurements and three different sets of ADD-values

**Fig. 4:**
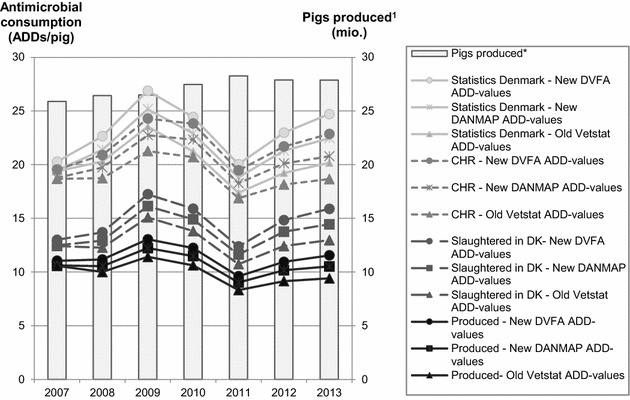
Annual antimicrobial consumption using four different population measurements and three different sets of ADD-values. Average annual antimicrobial consumption per pig calculated as number of Animal Daily Doses (ADDs)/pig using four different pig population measurements: (1) number of pigs according to Statistics Denmark, (2) number of pigs according to the Central Husbandry Register, (3) pigs slaughtered in Denmark per year and (4) pigs produced in Denmark per year and using three different sets of ADD-values: (a) the old VetStat ADD-values used in the Yellow Card initiative until the 29th of November 2014, (b) the DANMAP ADD-values used in the 2012 and 2013 DANMAP reports and (c) the Danish Veterinary and Food Administration’s ADD-values used in the Yellow Card initiative from the 30th of December 2014 and onwards. ^1^Pigs produced adjusted for an assumed 3.8 % mortality in exported growers

Twelve different ways of estimating the average annual antimicrobial consumption per pig arise when the four pig population measurements: (1) SD, (2) CHR, (3) SL-year and (4) PROD-year are combined with the three different sets of ADD-values: (a) old VetStat ADD-values, (b) new DANMAP ADD-values and (c) new DVFA ADD-values). All twelve are shown in Table 2 and graphically illustrated in Fig. 4.

**Table 2:** Annual antimicrobial consumption using four different population measurements and three different sets of ADD-values

Population	Statistics Denmark			Central Husbandry Register			Slaughtered in Denmark			Produced in Denmark		
ADD-values	Old VetStat	New DANMAP	New DVFA	Old VetStat	New DANMAP	New DVFA	Old VetStat	New DANMAP	New DVFA	Old VetStat	New DANMAP	New DVFA
2007	19.4	19.5	20.3	18.7	18.8	19.5	12.4	12.5	13.0	10.6	10.6	11.1
2008	20.3	21.4	22.6	18.7	19.7	20.9	12.3	12.9	13.7	10.0	10.5	11.2
2009	23.5	25.2	26.9	21.3	22.7	24.3	15.1	16.1	17.2	11.4	12.2	13.1
2010	21.2	22.9	24.4	20.7	22.3	23.8	13.8	14.9	15.9	10.6	11.5	12.2
2011	17.4	18.8	20.0	16.9	18.3	19.5	10.7	11.6	12.4	8.3	9.0	9.6
2012	19.2	21.3	23.0	18.2	20.1	21.7	12.4	13.8	14.8	9.2	10.2	10.9
2013	20.2	22.5	24.7	18.7	20.8	22.9	13.0	14.4	15.9	9.4	10.4	11.6

Average annual antimicrobial consumption per pig calculated as number of ADDs/pig using four different pig population measurements: (1) number of pigs according to Statistics Denmark, (2) number of pigs according to the Central Husbandry Register, (3) pigs slaughtered in Denmark per year and (4) pigs produced in Denmark per year and using three different sets of ADD-values: (a) the old VetStat ADD-values used in the Yellow Card initiative until the 29th of November 2014, (b) the DANMAP ADD-values used in the 2012 and 2013 DANMAP reports and (c) the Danish Veterinary and Food Administration’s ADD-values used in the Yellow Card initiative from the 30th of December 2014 and onwards

In 2013, the calculated consumption amounted to 15.9 ADDs/pig/year when using SL-year and the new DVFA ADD-values, 13.0 ADDs/pig/year when using SL-year and the old VetStat ADD-values, 11.6 when using PROD-year and the new DVFA ADD-values and 9.4 when using PROD-year and the old VetStat ADD-values. So, compared to the calculated results when using SL-year and the new DVFA ADD-values, the consumption in 2013 was 40.8 % lower when calculated based on PROD-year and the old VetStat ADD-values. This underlines how not including exported live pigs may highly alter the calculated results on antimicrobial usage, especially for a country such as Denmark with a substantial export of live pigs.

From 2007 to 2013, the antimicrobial consumption increased by 22 % when using either SD or SL-year as population measurement and the new DVFA ADD-values. However, if PROD-year was used as population measurement together with the old VetStat ADD-values, the consumption from 2007 to 2013 decreased by 10.9 %.

Following the announcement of the Yellow Card initiative, the antimicrobial consumption, calculated as ADDs/pig/year, decreased by ~20 % from 2010 to 2011 regardless of calculation method (Table 2). The increase in antimicrobial consumption from 2011 to 2013 was in contrast influenced by chosen calculation method with 13.1 % as the smallest increase observed (PROD-year/old VetStat ADD-values) and 28.3 % as the largest increase (SL-year/new DVFA ADD-values).

In a recent study by Taverne et al. [26], the Dutch pig antimicrobial consumption in 2012 was calculated with three different sets of ADD-values. Taverne et al. reported that the calculated antimicrobial consumption was highly affected by the chosen set of ADD-values for a single point in time. This result is in concurrence with the findings of this study, which additionally found that not only are the results affected when evaluating the consumption as one point in time, but also when evaluating trends in consumption over time.

This study only investigated ADD-values described for one country. However, recently a call has been made by the European Surveillance of Veterinary Antimicrobial Consumption consortium for a standardized set of ADD-values to be applied in all European Union member states when reporting veterinary antimicrobial usage [33]. However, this may be no easy task. In addition to differing within countries, ADD-values have also been reported to differ between countries [26], e.g. due to i) differences in theoretical foundations, or ii) products having been assigned an ADD-value in one country and not in another [26]. Additionally, Postma et al. [9] have reported differences in SPC stated dosages for products with identical active compound and administration route—both between and within countries. This highlights the fact that even though two sets of ADD-values from different countries may have identical theoretical foundations, e.g. both being based on product SPCs, there is no guarantee that the two sets will be identical.

When a set of common ADD-values have been established, it is still vital that the correct animal population is used as denominator, when attempting to assess true antimicrobial exposure. In a paper from 2013, Bondt et al. found that total sales data on all veterinary antimicrobials only gave a poor estimate of the actual antimicrobial exposure per animal species, as results were highly affected by the population demographics [8]. Bondt et al. [8] recommended to use census data i.e. number of animals present at any given time (in this paper the equivalent to SD or CHR data), rather than number of animals produced when estimating the population at risk. However, census data do not take turn-over of animals into account. An estimation of the antimicrobial exposure in numbers of ADDs will often be reported as “numbers of ADDs/pig/year” or as “numbers of ADDs/pig/day”. A calculated result based on CHR as population measurement of e.g. 20 ADDs/pig/year will often translate into 20 treatments per pig per year. However, this is highly misleading. In Denmark, a grower on average spends 7 weeks in the grower stable section, entering at 7 kg and leaving at 30 kg [34]. A herd with 500 growers registered in CHR will consequently have had roughly 3300 pigs through its facility in the course of 1 year, following the assumption that the herd stays empty for 1 week between each batch (53 weeks divided by 8 = 6.6; 6.6 multiplied by 500 registered pigs in CHR = 3300 actual pigs). If it is then assumed that the previously mentioned 20 ADDs/pig/year is based on data from growers, the actual number of average treatments per pig will be 3.03 (20 divided by 6.6). The fact that estimations of antimicrobial exposure based on SD or CHR data do not take productivity into account might also potentially lead to herds with a high production of pigs getting a higher consumption on paper when using CHR as a measurement for the population at risk. This even though the herd in fact may be using the same amount of antimicrobials per produced pig as a competing similar herd with a lower production. However, further studies are needed to discern the scope of this potential issue.

## Conclusions

The findings of this study clearly highlight that calculated national antimicrobial consumption is highly affected by chosen population measurement and applied ADD-values. When SD or SL-year were used as population measurement together with the new DVFA ADD-values, a 22 % increase was observed from 2007 to 2013 in the average annual antimicrobial consumption per pig, whereas the consumption in the same time span decreased with 11.3 % when using PROD-year as population measurement together with the old VetStat ADD-values. These quite substantial differences may partly be due to the large shift in the Danish pig industry’s production pattern with an increasing percentage of the produced pigs being exported to other countries before slaughter.

It is important to address the recent central change in ADD assignment regimen in Denmark, which occurred with the implementation of the two new sets of ADD-values by DANMAP and DVFA. Before 2012, the two main institutions to report the Danish pig antimicrobial consumption both utilized the exact same assignment method and the same set of ADD-values, which was located as a supplementary table in the VetStat database. However, as we now have two major national institutions who calculate Danish pig antimicrobial consumption based on different sets of ADD-values, it becomes imperative to ensure that the exact calculation method is stated both for the numerator (antimicrobial consumption in e.g. total kg of active compound or number of ADDs) and the denominator (population measurement) when reporting antimicrobial consumption, especially to avoid comparisons of numbers across years based on different calculation methods. In conclusion, it is essential to ensure transparency in all calculations used when reporting antimicrobial consumption, especially when wishing to evaluate the consumption over time or compare with other countries.

## References

[1] European Medicines Agency. Sales of veterinary antimicrobial agents in 28 EU/EEA countries in 2012. http://www.ema.europa.eu/docs/en_GB/document_library/Report/2014/10/WC500175671.pdf. Accessed 27 Oct 2015.

[2] Grave K, Torren-Edo J, Mackay D (2010). Comparison of the sales of veterinary antibacterial agent between 10 European countries. J Antimicrob Chemother.

[3] Callens B, Persoons D, Maes D, Laanen M, Postma M, Boyen F (2012). Prophylactic and metaphylactic antimicrobial use in Belgian fattening pig herds. Prev Vet Med..

[4] Merle R, Hajek P, Käsbohrer A, Hegger-Gravenhorst C, Mollenhauer Y, Robanus M (2012). Monitoring of antibiotic consumption in livestock: a German feasibility study. Prev Vet Med..

[5] Netherlands Veterinary Medicines Authority. Usage of antibiotics in livestock in the Netherlands in 2012. http://www.autoriteitdiergeneesmiddelen.nl/Userfiles/pdf/sda-report-usage-of-antibiotics-in-livestock-in-the-netherlands-in-2012-July-2013.pdf. Accessed 31 Mar 2016.

[6] Chauvin C, Madec F, Guillemot D, Sanders P (2001). The crucial question of standardisation when measuring drug consumption. Vet Res.

[7] Timmerman T, Dewulf J, Catry B, Feyen B, Opsomer G, Kruif Ad (2006). Quantification and evaluation of antimicrobial drug use in group treatments for fattening pigs in Belgium. Prev Vet Med..

[8] Bondt N, Jensen VF, Puister-Jansen LF, van Geijlswijk IM (2013). Comparing antimicrobial exposure based on sales data. Prev Vet Med..

[9] Postma M, Sjölund M, Collineau L, Lösken S, Stärk KD, Dewulf J (2015). Assigning defined daily doses animal: a European multi-country experience for antimicrobial products authorized for usage in pigs. J Antimicrob Chemother.

[10] Stege H, Bager F, Jacobsen E, Thougaard A (2003). VETSTAT—the Danish system for surveillance of the veterinary use of drugs for production animals. Prev Vet Med..

[11] Doe J. Current antimicrobial reports. In: VetStat, a register on prescription-only medicinal products for animals. Ministry of environment and food, the Danish veterinary and food administration. 2015. http://www.foedevarestyrelsen.dk/Leksikon/Sider/VetStat.aspx. Accessed 20 Oct 2015.

[12] Statens Serum Institut, National Veterinary Institute, National Food Institute. DANMAP 2013—use of antimicrobial agents and occurrence of antimicrobial resistance in bacteria from food animals, food and humans in Denmark. http://www.danmap.org2014. Accessed 5 Oct 2015.

[13] Jensen VF, de Knegt L, Andersen VD, Wingstrand A (2014). Temporal relationship between decrease in antimicrobial prescription for Danish pigs and the “Yellow Card” legal intervention directed at reduction of antimicrobial use. Prev Vet Med..

[14] Alban L, Dahl J, Andreasen M, Petersen J, Sandberg M (2013). Possible impact of the “Yellow Card” antimicrobial scheme on meat inspection lesions in Danish finisher pigs. Prev Vet Med..

[15] Jensen VF, Jacobsen E, Bager F (2004). Veterinary antimicrobial-usage statistics based on standardized measures of dosage. Prev Vet Med..

[16] Statens Serum Institut, National Veterinary Institute, National Food Institute. DANMAP 2012- DADD description. http://www.danmap.org/~/media/Projekt%20sites/Danmap/DANMAP%20reports/DANMAP%202012/DANMAP%202012%20DADD%20description.ashx. Accessed 6 Oct 2015.

[17] Doe J. ADD and antimicrobial consumption. The Ministry of Food, Agriculture and Fisheries of Denmark. http://www.foedevarestyrelsen.dk/Leksikon/Sider/ADD-og-antibiotikaforbrug-i-kv%C3%A6g--og-svinebes%C3%A6tninger.aspx. Accessed 10 Jun 2015.

[18] Statens Serum Institut, National Veterinary Institute, National Food Institute. DANMAP 2009—use of antimicrobial agents and occurrence of antimicrobial resistance in bacteria from food animals, food and humans in Denmark. http://www.danmap.org/downloads/reports.aspx. Accessed 5 Oct 2015.

[19] Doe J. Legal Act number 178: Legal Act on limit values for antimicrobial consumption in cattle and swine herds. (In Danish: Bekendtgørelse om grænseværdier for antibiotikaforbrug i kvæg- og svinebesætninger). Retsinformation. 2015. https://www.retsinformation.dk/Forms/R0710.aspx?id=161940. Accessed 1 Oct 2015.

[20] Ferech M, Coenen S, Malhotra-Kumar S, Dvorakova K, Hendrick E, Suetens C (2006). European Surveillance of Antimicrobial Consumption (ESAC): outpatient antibiotic use in Europe. J Antimicrob Chemother.

[21] MacKenzie FM, Gould IM, Gould IM, Van Der Meer JWM (2005). Quantitative measurement of antibiotic use. Antibiotic Policies.

[22] Doe J. Statistics—pigmeat. Danish Agriculture and Food Council. 2012, http://lf.dk/Tal_og_Analyser/Aarstatistikker/Statistik_svin/Tidligeres_statistikker.aspx. Accessed 30 Mar 2016.

[23] Doe J. Statistics–pigmeat. Danish Agriculture and Food Council. 2014, http://lf.dk/Tal_og_Analyser/Aarstatistikker/Statistik_svin/Tidligeres_statistikker.aspx. Accessed 30 Jun 2015.

[24] Dupont N, Stege, H, Toft, N, Andreasen, M, Enøe, C. Reporting antibiotic consumption for Danish pigs—effect of denominator. In: Park BK (ed) Proceedings from the International Pig Veterinary Society Congress 2011; Jeju, South Korea. p. 216.

[25] González SM, Steiner A, Gassner B, Regula G (2010). Antimicrobial use in Swiss dairy farms: quantification and evaluation of data quality. Prev Vet Med..

[26] Taverne FJ, Jacobs JH, Heederik DJJ, Mouton JW, Wagenaar JA, van Geijlswijk IM (2015). Influence of applying different units of measurement on reporting antimicrobial consumption data for pig farms. BMC Vet Res..

[27] Larsen M. Pigs—administrative information about the Statistical Product. In: Production animals. Statistics Denmark. 2014. http://www.dst.dk/en/Statistik/dokumentation/Declarations/pigs.aspx. Accessed 27 Nov 2014.

[28] Doe J. Legal Act number 1237: Legal Act on registration of herds in CHR. (In Danish: Bekendtgørelse om registrering af besætninger I CHR). Retsinformation. 2015. https://www.retsinformation.dk/Forms/R0710.aspx?id=158819. Accessed 1 Oct 2015.

[29] Doe J. Legal Act number 1383: Legal Act on marking, registration and moving cattle, pigs, sheep and goats. (In Danish: Bekendtgørelse om mærkning, registering og flytning af kvæg, svin, får og geder). Retsinformation. 2015. https://www.retsinformation.dk/Forms/R0710.aspx?id=166914. Accessed 2 Oct 2015.

[30] Doe J. Statistics—pigmeat. Danish Agriculture and Food Council. 2015, http://lf.dk/Tal_og_Analyser/Aarstatistikker/Statistik_svin/Tidligeres_statistikker.aspx. Accessed 30 Jun 2015.

[31] Larsen M. Landsgennemsnit for produktivitet i svineproduktionen 2013. SEGES—Danish Agriculture and Food Council. 2014. http://vsp.lf.dk/Publikationer/Kilder/Notater/2014/1422.aspx. Accessed 1 Jun 2015.

[32] Marquer P, Rabade T, Forti R. Pig farming in the European Union: considerable variations from one Member state to another. In: Pig farming sector—statistical portrait 2014. EUROSTAT. 2014. http://ec.europa.eu/eurostat/statistics-explained/index.php/Pig_farming_sector_-_statistical_portrait_2014. Accessed 30 Oct 2015.

[33] European Medicines Agency. Revised ESVAC reflection paper on collecting data on consumption of antimicrobial agents per animal species, on technical units of measurement and indicators for reporting consumption of antimicrobial agents in animals. http://www.ema.europa.eu/docs/en_GB/document_library/Scientific_guideline/2012/12/WC500136456.pdf. Accessed 30 Mar 2016.

[34] SEGES – Danish Pig Research Centre, Danish Agriculture and Food Council. Smågrisestald. http://vsp.lf.dk/Viden/Stalde/Staldindretning/Smaagrisestald.aspx. Accessed 30 Mar 2016.

